# The dynamics of disease in a metapopulation: The role of dispersal range

**DOI:** 10.1016/j.jtbi.2017.01.037

**Published:** 2017-04-07

**Authors:** Ace R. North, H. Charles J. Godfray

**Affiliations:** Department of Zoology, University of Oxford, South Parks Road, Oxford OX1 3PS, UK

**Keywords:** Metapopulation, Disease, Spatial moments, Correlated landscape, Dispersal kernel

## Abstract

The establishment and spread of a disease within a metapopulation is influenced both by dynamics within each population and by the host and pathogen spatial processes through which they are connected. We develop a spatially explicit metapopulation model to investigate how the form of host and disease dispersal jointly influence the probability of disease establishment and invasion. We show that diseases are more likely to establish if both the host and the disease tend to disperse locally, since the former leads to the spatial aggregation of host populations in the environment while the latter facilitates the pathogen's exploitation of this spatial pattern. In contrast, local pathogen dispersal is likely to reduce the probability of subsequent disease spread because it increases the spatial segregation of infected and uninfected populations. The effects of local dispersal on disease dynamics are less pronounced when the pathogen spreads through the movement of infected hosts and more pronounced when pathogen dispersal is independent (for example through airborne viruses) though the details of host and pathogen biology can be important. These spatial effects tend to be more pronounced if the sites available for host occupation are themselves spatially aggregated.

## Highlights

• We explore disease spread in spatially structured metapopulations.
• Local host dispersal facilitates disease invasion but hinders disease persistence.
• These effects are strongest for diseases that spread independently of the host.
• They are also stronger in landscapes where the habitat sites are clumped in space.

## 1 Introduction

Many species are distributed across the landscape in metapopulations, ensembles of populations connected by dispersal which persist despite the limited lifespan of any of their constituents (Hanski and Gaggiotti, 2004, Clobert et al., 2004). Diseases can move through metapopulations and the dispersal behaviour of the host (and of the pathogen if independent) can simultaneously influence metapopulation structure and disease dynamics in complex and interacting ways. Understanding this issue is of practical importance to managing diseases of humans, livestock and crops (e.g. Ferguson et al., 2001; Keeling et al., 2001; Hufnagel et al., 2004). It is also of interest to conservation biologists who seek to understand how manipulating the connectivity of an environment (for example by constructing habitat corridors) will alter the risk of diseases entering endangered metapopulations (e.g. Hess, 1996; Gog et al., 2002; McCallum and Dobson, 2002).

Most models of diseases in metapopulations have assumed either that dispersing host individuals, and pathogen propagules (where the pathogen can disperse independently of the host), are distributed evenly across habitat sites (e.g. Hess, 1996; McCallum and Dobson, 2002; Gog et al., 2002; Harding et al., 2012) or are restricted to move between neighbouring sites in a network (e.g. Cross et al., 2005, Cross et al., 2007). The former assumption has allowed investigation of the dispersal *rate*, i.e. the propensity to disperse. For instance, Hess (1996) took a classical ecological (Levins) metapopulation and derived conditions for the invasion of a disease that increases the risk of population extinction. Disease incidence increases with dispersal rate in this model for two reasons. First, the movement of infected hosts spreads the disease to uninfected parts of the metapopulation. Second, dispersal enables the colonisation of unoccupied sites, and thereby increases the number of susceptible populations where the disease may establish. Building on Hess's model (Hess, 1996), other studies have considered the consequences of incorporating multiple host species (Gog et al., 2002, McCallum and Dobson, 2002, Jesse et al., 2008, Harding et al., 2012), populations that through the build up of immunity become resistant to further infections (McCallum and Dobson, 2002, Jesse et al., 2008), Allee effects (Harding et al., 2012), and explicit local population dynamics (Jesse et al., 2008). These studies generally support the conclusion that disease incidence increases with dispersal rate although greater dispersal may lead to epidemics of shorter duration (Jesse et al., 2008). Since dispersal leads to both new patch colonisation and to disease spread in these studies, the overall effects on metapopulation persistence are complex and dependent on the detailed biology of the system.

The assumption of restricted movements has helped the investigation of how epidemics are influenced by the spatial pattern of disease spread. In a single spatially-structured population, the distribution of a disease will be clustered if interactions are spatially limited, so that infected individuals tend to have more contact with other infected individuals than with susceptible individuals (Keeling, 1999). Similarly, in a metapopulation, infected populations will tend to become clustered if the migration of infection between populations is spatially limited (Watts et al., 2005, Cross et al., 2005, Cross et al., 2007). This factor may slow or even prevent epidemics from occurring, suggesting that the range of dispersal may be at least as important as the rate of dispersal to pathogen dynamics in structured host populations (Keeling, 1999, Brown and Bolker, 2004, Watts et al., 2005). The significance of spatial structure depends on both disease and host population characteristics (Brown and Bolker, 2004, Cross et al., 2005, Cross et al., 2007, Watts et al., 2005, Colizza and Vespignani, 2008). For instance, a disease with low virulence but a long infectious period will be more able to invade a structured metapopulation than a similar disease that is more virulent but with a shorter infectious period (Cross et al., 2005). Models investigating these factors have tended to assume that the spatial structure of the host population is independent of the biology of the host. This assumption is fully justified for host populations with static and well understood spatial distributions, such as human and livestock populations that are a focus of much epidemiological research. A great many natural populations, however, inhabit highly fragmented landscapes and undergo complex spatial dynamics (Hanski and Gaggiotti, 2004). The role of dispersal is less clear where host movement not only underlies disease spread, but also the structure of the metapopulation.

Here we ask how dispersal *distance* influences disease invasion in spatially structured metapopulations. We pay particular attention to two limiting cases. In the first the pathogen spreads through the environment solely through the movement of infected hosts. In the second the pathogen disperses independently of the host, for example through the movement of airborne propagules. Note that in both cases host dispersal indirectly affects pathogen spread because of its influence on the underlying structure of the host metapopulation. Of course, both processes can occur simultaneously, yet by considering these limiting cases separately we are able to explore the different ways host dispersal range (and in the second case also pathogen dispersal range) influence disease dynamics.

We analyse a spatially explicit version of the host-pathogen model first described by Hess (1996). Our host model is based on a spatially explicit and stochastic version of Levins' (1969) classical metapopulation, where potential subpopulation sites may be randomly distributed or clumped in space as described by a spatial covariance function. Sites may be permanent (though sometimes unoccupied), or they may appear and disappear dynamically. The dynamics of this type of metapopulation in the absence of disease have been studied by Ovaskainen and Cornell, 2006a, Cornell and Ovaskainen, 2008 who show that they can be modelled by a set of equations describing changes in the mean, spatial variance and higher moments. First-order perturbation expansions can be used to explore how the introduction of local interactions causes the behaviour to depart from the classical metapopulation case. We expand this approach to the host-pathogen metapopulation to derive approximate expressions for the metapopulation disease establishment threshold $R_{*}$, which is the equivalent of $R_{0}$ in classical epidemiology (Ball et al., 1997), as well as the invasion dynamics and the equilibrium pathogen incidence. The accuracy of the approximations is tested using simulations.

## 2 Model and methods

In this section we first present the model that describes the spatial and temporal distribution of potential sites that can be occupied by the host population, before going on to define how populations interact across the environment. Finally, we outline the derivation of the first order moment equations that we use to study the behaviour of the model. More formal details are given in the Supplementary material.

### 2.1 Site dynamics

Following Cornell and Ovaskainen (2008), we suppose that potential sites for host populations are created in clusters and so can be spatially correlated. Cluster centres are assumed to occur randomly in space with the number of sites per cluster a Poisson-distributed random variable with mean *ν*. The position of any particular site with respect to the cluster centre is determined by a spatially symmetric kernel $K_{\lambda }\left(\rho \right)$ where *ρ* is the distance from the centre of the cluster and λ is the kernel width. Environments are assumed to be fixed or dynamic. In the latter case clusters arise at rate *α* and sites disappear at rate β so that the equilibrium density of sites is $\frac{\alpha \nu }{\beta }$.

### 2.2 Site type transition rates

There are three classes of site: empty (*E*), occupied by susceptible hosts (*S*) and occupied by infected hosts (*I*). Susceptible and infected populations can go extinct at rates *μ*_*S*_ and *μ*_*I*_ respectively and produce migrants at rates *m*_*S*_ and *m*_*I*_ whose movements are described by two spatially symmetric kernels *D*_*S*_(*ρ*) and *D*_*I*_(*ρ*) where *ρ* is distance from the original site. The rate at which a particular empty site is colonised by a population of susceptible hosts is $m_{S}\sum_{i\in S}D_{S}\left(\rho_{i}\right)$ where the summation is over all susceptible sites in the environment and $\rho_{i}$ is the distance from susceptible site *i* to the focal site. An equivalent expression, $m_{I}\sum_{i\in I}D_{I}\left(\rho_{i}\right)$, describes colonisation of empty sites by infected hosts, and this same expression scaled by a factor *b* describes the rate at which susceptible sites become infected by the movement of infected hosts. The parameter *b* thus describes the infectiousness of a disease relative to the ability of infected individuals to colonise empty sites, and can be greater or less than one.

Finally we assume that infected sites produce pathogen propagules at rate *m*_*P*_ whose spatial dispersal is described by a further kernel, *D*_*P*_(*ρ*). With these assumptions the five transition rates amongst the three types of site can be given by the expressions in Table 1. As is described more formally in the Supplementary material, the shapes of the three dispersal kernels are governed by parameters *δ*_*S*_, *δ*_*I*_ and *δ*_*P*_. A low value of *δ*_*S*_ means that the *m*_*S*_ migrants originating from susceptible sites tend to remain in the vicinity of their original site, and a high value that they disperse further (with equivalent interpretations for *δ*_*I*_ and *δ*_*P*_). In the rest of the paper we shall assume that infections alter the migration rate $\left(m_{S}\neq m_{I}\right)$ but that the dispersal kernels are the same (*D*_*S*_(*ρ*)=*D*_*I*_(*ρ*)).

### 2.3 Population dynamics

Over time the relative densities of the three site types will change (and hence we write them as a function of time: *E*(*t*), *S*(*t*) and *I*(*t*)). Because of local interactions we know that the metapopulation will tend towards non-random spatial patterns of site occupancy that will in turn influence the ensemble dynamics. These patterns can be described by a series of spatial moment functions of which the first describes average densities (denoted by $\overset{̅}{S}\left(t\right)$ etc. or just $\overset{̅}{S}$ when the dependence on time is clear) and the second the spatial covariances. For example, we can define $G_{\mathrm{SI}}\left(\rho ,t\right)$ to be the covariance at time *t* in the distribution of susceptible and infected sites separated by distance ρ (and similarly for other site combinations). In general, the transition rates between two particular site types will depend on their spatial covariance and the appropriate dispersal kernel (as defined in the previous section). Specifically, define $\Gamma_{\mathrm{YZ}}^{X}\left(t\right)$ to be the spatial convolution of the spatial covariance function between site types *Y* and *Z* and the dispersal kernel *X*. For example,

(1) $$\Gamma_{SE}^{S}\left(t\right)=\int G_{SE}\left(\rho ,t\right)D_{S}\left(\rho \right)d\rho ,$$

describes the net colonisation of empty patches by susceptible hosts which is influenced by both spatial structure (the degree to which empty sites tend to occur or not occur near susceptible sites) and the dispersal kernel for susceptible hosts. As is derived formally in the Supplementary material we can then write equations for the change in the average density of the patch types (superscript bars denote averages) that make up the system,

(2) $$\begin{array}{c} \frac{d\overset{}{\overset{¯}{S}}}{\mathrm{dt}}=\left[m_{S}\overset{}{\overset{¯}{S}}\overset{}{\overset{¯}{E}}-m_{I}b\overset{}{\overset{¯}{S}}\overset{}{\overset{¯}{I}}-m_{P}\overset{}{\overset{¯}{S}}\overset{}{\overset{¯}{I}}-\left(\mu_{S}+\beta \right)\overset{}{\overset{¯}{S}}\right]+\left[m_{S}\Gamma_{\mathrm{SE}}^{S}-m_{I}{b\Gamma }_{\mathrm{SI}}^{I}-m_{P}\Gamma_{\mathrm{SI}}^{P}\right],\\ \frac{d\overset{}{\overset{¯}{I}}}{\mathrm{dt}}=\left[m_{I}\overset{}{\overset{¯}{I}}\overset{¯}{\overset{}{E}}+m_{I}b\overset{}{\overset{¯}{S}}\overset{}{\overset{¯}{I}}+m_{P}\overset{}{\overset{¯}{S}}\overset{}{\overset{¯}{I}}-\left(\mu_{I}+\beta \right)\overset{}{\overset{¯}{I}}\right]+\left[m_{I}\Gamma_{\mathrm{IE}}^{I}+m_{I}{b\Gamma }_{\mathrm{SI}}^{I}+m_{P}\Gamma_{\mathrm{SI}}^{P}\right],\;\frac{d(\overset{}{\overset{¯}{E}}+\overset{¯}{\overset{}{S}}+\overset{¯}{\overset{}{I}})}{\mathrm{dt}}=\alpha \nu -\beta (\overset{}{\overset{¯}{E}}+\overset{¯}{\overset{}{S}}+\overset{}{\overset{¯}{I}}). \end{array}$$

Note that in the first two of Eq. (2), the terms in the first square brackets summarise all the effects that are independent of spatial structure and those in the second the contribution of spatial structure.

Unfortunately these equations do not fully specify the system. Further expressions for changes in the variance and covariance functions are needed, but just as equations for changes in the mean incorporate second moment terms, those for second moments include third moments and so on *ad infinitum*. One solution is to take a ‘moment closure’ and either ignore all terms above a given order or replace them by a function of lower moments (e.g. Bolker and Pacala, 1997). In this paper we take a second approach and apply a perturbation expansion to the spatial moments around a mean field limit. The formal derivation is given in the Supplementary material but in brief power series expansions of the mean density of each site type are found, for example,

(3) $$\overset{̅}{S}\left(t\right)={\overset{̅}{S}\left(t\right)}^{\left(0\right)}+\epsilon {\overset{̅}{S}\left(t\right)}^{\left(1\right)}+\epsilon^{2}{\overset{̅}{S}\left(t\right)}^{\left(2\right)}\ldots ,$$

where the superscripts (0), (1), (2) denote zero'th, first, second order components etc., and ϵ is the perturbation parameter which becomes small as the different environment and dispersal kernels broaden. This is implemented by allowing the kernel width parameters $\delta_{S},\delta_{I},\delta_{P}$ and λ to be implicitly scaled by 1/√ϵ, by defining, for example, $\delta_{S}=\delta_{S}^{\prime }/\surd \epsilon$. The effect of reducing ϵ is thus to increase these four parameters simultaneously without altering their relative proportions.

We can obtain a closed set of equations up to any given order of the perturbation expansion because higher spatial moments do not contribute to lower order terms of the expansion. For example, the second moment (covariance) functions have no zero’th order components and in general the *x*'th moment has no *x*-2 or lower order components (Ovaskainen and Cornell, 2006b). The terms of order zero reveal the behaviour of the spatially implicit version of the model which assumes an effectively infinite migration range. The first order and above terms reveal the deviation from the mean field model due to the effects of local interaction and stochasticity in colonisation and extinction events as the system is “perturbed” towards the full spatially explicit model. Here we restrict our analysis to the zero’th and first orders. We test the effectiveness of the approximation by simulation and find that it enables us to predict the roles of spatial interaction and stochasticity some way from the mean-field limit.

## 3 Results

### 3.1 The absence of disease

The disease free model has been studied by Cornell and Ovaskainen (2008) and the limiting case of no local spatial interactions is a classical Levins metapopulation. Several results are useful for what follows. In the non-local case the equilibrium density of occupied patches is

(4) $${\overset{̅}{S}}^{*}=1-\frac{\mu_{S}+\beta }{m_{s}}.$$

Thus the metapopulation exists as long as $m_{s}>\mu_{S}+\beta$.

If migration is a local process, the metapopulation will tend towards a spatially aggregated pattern of occupancy. This spatial structure influences the rate at which vacant sites are colonised, and the extent of patch occupancy will thus differ from the value given above (Ovaskainen and Cornell, 2006a, Cornell and Ovaskainen, 2008). In simple uncorrelated environments (i.e., the distribution of sites is random), the rate of colonisation of new sites is lower as more migrants arrive at neighbouring sites, which tend to be occupied, rather than at distant sites, which are more likely to be vacant. Hence aggregation acts to reduce the equilibrium density of occupied sites. In correlated environments, however, potential population sites are aggregated and local migration takes advantage of this aggregation (‘habitat association’, Bolker, 2003). These two competing factors may combine to either increase or decrease site occupancy compared to the non-spatial model, depending on the extent of environmental correlation and the spatial scales at which it occurs. If the environment is dynamic rather than static (β>0), the influence of both spatial factors is reduced, because the loss and creation of sites across the environment acts to reduce the extent of population aggregation (Cornell and Ovaskainen, 2008).

### 3.2 Disease establishment

The disease is able to establish in the metapopulation if the number of new sites colonised by an initial infected site is greater than one ( $R_{*}$>1). This condition can be derived from Eq. (2b) as *I*(*t*)→0 and where the average density of the susceptible sites is assumed to be at its equilibrium, ${\overset{̅}{S}}^{*}$,

(5) $$R_{*}=\frac{1}{\mu_{I}+\beta }\left(\left[m_{I}(1-{\overset{̅}{S}}^{*}){+m}_{I}b{\overset{̅}{S}}^{*}{+m}_{P}{\overset{̅}{S}}^{*}\right]+\left[\underset{\overset{̅}{I}\to 0}{\lim}\frac{m_{I}\Gamma_{\mathrm{IE}}^{I}{+m}_{I}{b\Gamma }_{\mathrm{SI}}^{I}+m_{P}\Gamma_{\mathrm{SI}}^{P}}{\overset{̅}{I}}\right]\right).$$

#### 3.2.1 No local spatial interactions

Consider first the case where spatial processes have no effect on disease spread (the second square bracket term in Eq. (5) is zero). $R_{*}$ is then simply the average lifetime of a site ($1/\left[\mu_{I}+\beta \right])$ multiplied by the sum of the rates at which new infected sites are produced through (i) colonisation of empty sites, (ii) infection of susceptible sites by dispersing individuals, and (iii) infection of susceptible sites by dispersing pathogen propagules (the three terms in the first square bracket term in Eq. (5)).

Suppose that the disease is only spread by the movement of infected hosts (*m*_*p*_=0). The limiting case of the disease negatively affecting patch longevity ($\mu_{S}<\mu_{I}$) but not migration (*m*_*S*_=*m*_*I*_) was studied by Hess (1996) who showed that the persistence of the disease required $m_{s}>\mu_{S}+\beta +\frac{\mu_{I}-\mu_{S}}{b}$ which is a higher migration rate than that required for persistence in the absence of disease. This implies that very fragile metapopulations with migration rates just above the existence threshold are unlikely to be invaded by a disease and then driven to extinction.

Now assume the disease is only spread by the dispersal of pathogen propagules (*m*_*I*_=0). Disease persistence requires $m_{s}>\left(\mu_{S}+\beta \right)\left(\frac{m_{P}}{m_{P}-\left(\mu_{I}+\beta \right)}\right)$ which again requires higher host dispersal than for population persistence without disease, similarly implying that fragile metapopulations will not be invaded and driven extinct by the disease.

#### 3.2.2 Spatially structured metapopulations

We now ask how the initial spread of the disease is affected by metapopulation structure. To do this we explore the two limiting cases of (i) disease spread by the movement of infected hosts and (ii) disease spread by the dispersal of infectious propagules independent of host movement. In the first case, the initial disease incidence can occur either in an empty population site or one containing susceptibles, in the second it can only occur in a susceptible population. As shown more fully in the Supplementary material, in the limit when the infection is rare the convolutions involving the covariance terms *G*_*IE*_ and *G*_*SE*_ in Eq. (5) can be approximated using the equilibrium values of *G*_*SE*_ and *G*_*SS*_ respectively. These covariances can be estimated from presence-absence data across habitat sites, though the migration kernels are also required to construct the convolutions. In the Supplementary material we derive a perturbation expansion of the rate of disease increase when rare and here study it to first order,

(6) $$R_{*}\approx R_{*}^{\left(0\right)}+\epsilon R_{*}^{\left(1\right)},$$

using simulations to judge the accuracy of the approximation. Note in particular that when ${R_{*}}^{\left(0\right)}$ is close to unity, whether the pathogen will invade depends only on the second term except where the perturbation parameter (ϵ) is small which reflects the case of low spatial correlations and non-local dispersal. In what follows we largely set ${R_{*}}^{\left(0\right)}$ to unity and explore the effects of the parameters that determine the higher order terms, and unless stated assume static rather than dynamic environments. We draw five major conclusions from our investigations.

##### 3.2.2.1 Short range host migration can facilitate disease establishment in spatially uncorrelated environments

**Fig. 1:**
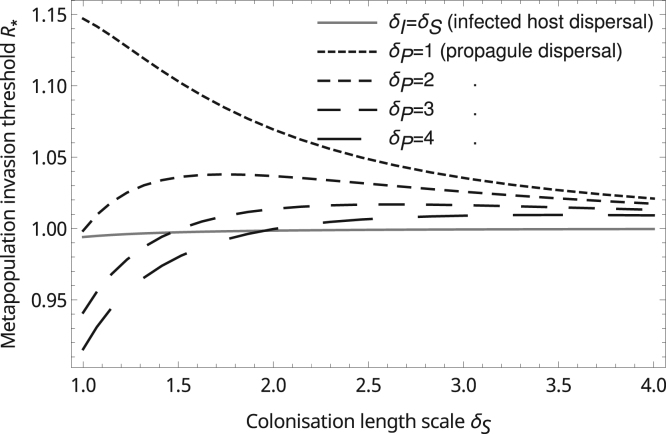
The effect of host migration scale on the metapopulation invasion threshold $R_{*}$. If the disease spreads by dispersing pathogen propagules (dashed lines), low values of $\delta_{P}$ indicate the dispersal is local. For all the examples, the sites within the metapopulation have an uncorrelated and static distribution with average density 1, and the mean-field model ($\delta_{S}=\delta_{I}=\delta_{P}=\infty$) predicts $R_{*}=1$. The parameters are $m_{I}=0.5,b=1.\dot{3},m_{P}=0$ for the infected host dispersal example, $m_{I}=0,m_{P}=1$ for the propagule dispersal cases and remaining parameters are $m_{S}=1,\mu_{S}=0.4,\mu_{I}=0.6$ for all cases. All examples use Gaussian dispersal kernels $D_{X}\left(\rho \right)=e^{-{\left(\frac{\rho }{\delta_{X}}\right)}^{2}}/\left(\pi \delta_{X}^{2}\right)\;\mathrm{for}\;X\in \left\{S,I,P\right\}$.

A disease that spreads by dispersing pathogen propagules can only establish an infection in a site occupied by susceptibles. If the propagules tend to be dispersed locally, the nearby presence of other susceptible populations, as occurs in spatially structured metapopulations, increases the chance of disease establishment given an initial rare infection (Fig. 1). This effect does not occur if the propagules are dispersed over large distances (*δ*_*P*_ is large) as then the disease fails to exploit its initial proximity to susceptible populations. Indeed, such a disease will be less likely to establish because if host dispersal is limited, at equilibrium it will occupy a smaller fraction of patches (as discussed above). The advantage also does not occur if the initial infection is as likely to appear in an area with comparatively few hosts as in a susceptible host cluster, which may be the case for diseases that are spread through the movement of infected hosts. The positive effect of host spatial structure on disease establishment is greatest when host and pathogen dispersal kernels both have small variances (*δ*_*S*_ and *δ*_*P*_ are small).

##### 3.2.2.2 Correlated environments can facilitate disease establishment

**Fig. 2:**
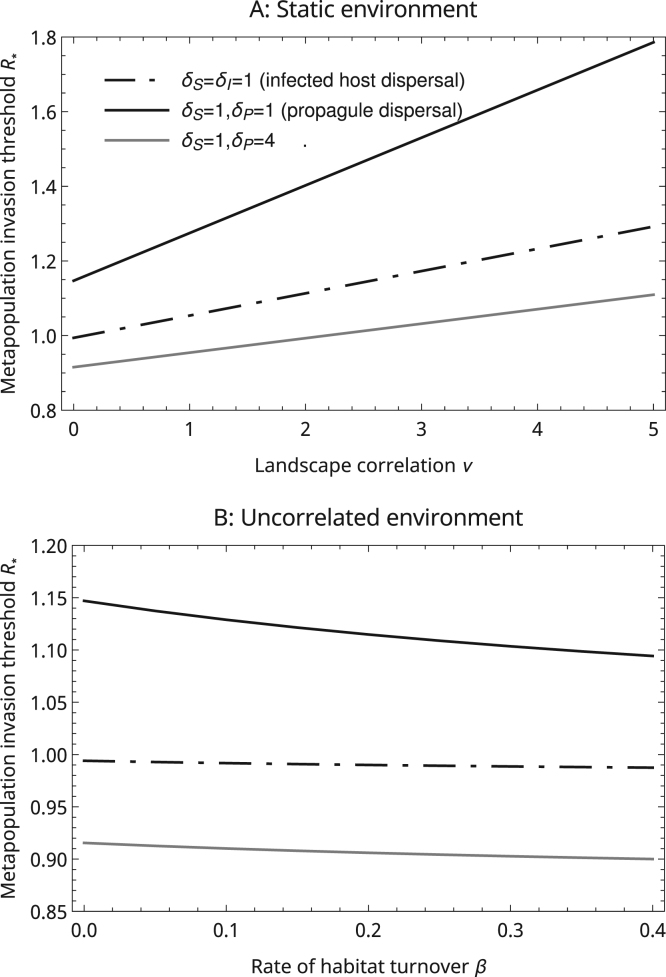
Effects of underlying environmental structure on the metapopulation invasion threshold $R_{*}$. To approximate static environments (A) we set $\beta =10^{-8}$ and to approximate uncorrelated environments (B) we set $\nu =10^{-8}$. In each case α is varied along the x-axis so that the average density of sites, $\frac{\alpha \nu }{\beta }$, is always 1. The demographic parameters are the same as in Fig. 1 so that the non-local model predicts $R_{*}=1$, and the landscape kernel is Gaussian with length scale λ=1 ($K_{\lambda }\left(\rho \right)=e^{-\rho^{2}}/\pi$).

If the sites within a metapopulation are spatially correlated then susceptible populations will be both more aggregated and, as discussed above, at equilibrium occupy a greater fraction of sites compared to equivalent uncorrelated environments. The increased aggregation facilitates the spread of diseases that spread through the short-ranged dispersal of pathogen propagules, while the greater frequency of occupied sites in the metapopulation benefits diseases that spread by infecting susceptible populations rather than through infected individuals colonising empty sites (Fig. 2A).

##### 3.2.2.3 Disease establishment is easier in static than dynamic metapopulations

In a dynamic metapopulation new empty sites are constantly being created and existing sites, irrespective of their occupation status, are being destroyed. This will tend to reduce the degree to which susceptible host populations are aggregated, especially in uncorrelated environments. Adjusting for the direct effects of site turnover (which occurs at rate β on ${\overset{̅}{S}}^{{\left(0\right)}^{*}}$ (by keeping $\beta +\mu_{S}$ constant) and on $R_{*}^{\left(0\right)}$ (by keeping $\beta +\mu_{I}$ constant)), we find that these effects combine so that $R_{*}$ tends to belower in dynamic landscapes. However, the net effect tends to be small, especially for diseases that spread by infected host dispersal (Fig. 2B).

##### 3.2.2.4 Spatial structure affects the tradeoff between infected dispersal and infectivity

**Fig. 3:**
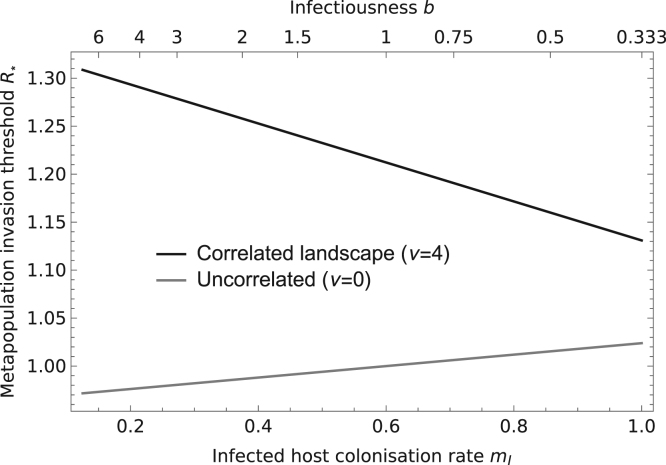
The relative importance of infectiousness (b) and migration ($m_{I}$) for the spread of a disease that is transmitted by the movement of infected hosts. The non-local model predicts $R_{*}=1$ for each combination of b and $m_{I}$ specified by the upper and lower scales on the x-axis. Other parameters are the same as in Fig. 1.

For diseases that are spread by the movement of infected individuals, different combinations of dispersal rate (*m*_*I*_) and infectiousness (*b*) can give rise to the same value of $R_{*}^{\left(0\right)}$. Introducing spatial structure alters the form of this trade-off due to its effects on the frequency and distribution of infected sites in the metapopulation. In uncorrelated environments, spatial structure reduces the fraction of sites that are occupied by susceptible populations, so diseases are more likely to spread if they have a high dispersal rate which promotes the colonisation of empty patches. In correlated environments, if the aggregation of sites is sufficient to increase the fraction of occupied sites in the absence of disease, the reverse is true: diseases are more likely to spread if they are more infectious (Fig. 3).

##### 3.2.2.5 First order perturbation theory reliably predicts $R_{*}$ unless interaction scales are small

**Fig. 4:**
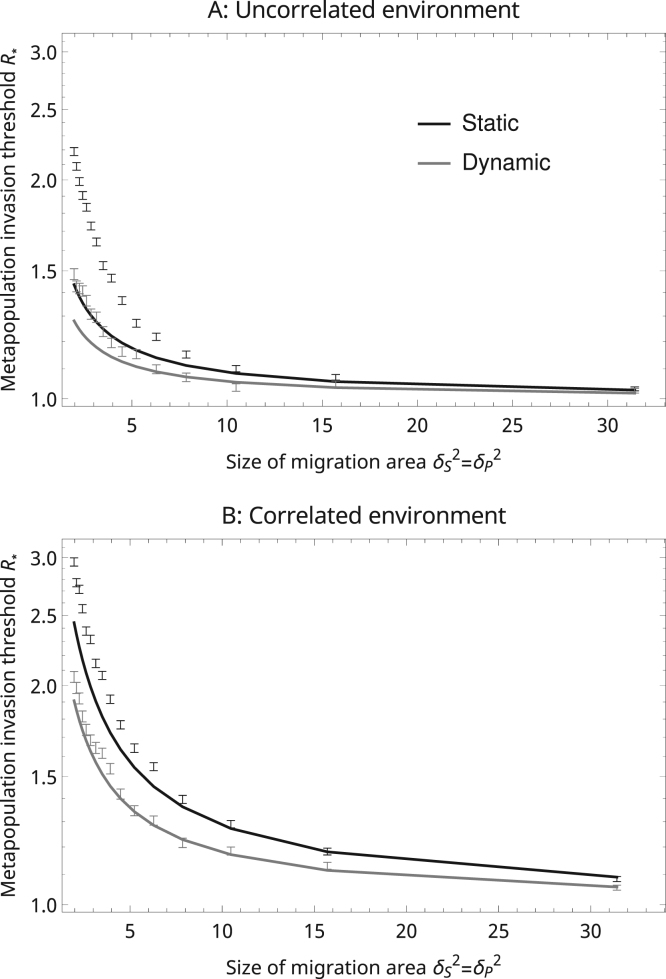
Comparison of the first order approximations (lines) to finite-space simulations of a disease that spreads by the dispersal of pathogen propagules independent of the host. Parameters are the same as in Fig. 1, with equal host and disease length scales for each position on the x-axis ($\delta_{S}=\delta_{P}=\lambda$). The dispersal kernels $D_{S}\left(\rho \right)$ and $D_{P}\left(\rho \right)$ are 'top-hat' ($D_{X}\left(\rho \right)=\frac{1}{\pi \delta_{X}^{2}}$ if ${\rho <\delta }_{X}$ or 0 otherwise) and the landscape kernel is Gaussian. The error bars show ±1 standard error either side of the simulation means.

We evaluated the performance of the first order approximation to $R_{*}$ away from the mean-field limit through comparison with simulations for a disease that spreads by the dispersal of pathogen propagules (the case where spatial effects are most significant, Fig. 1). The simulations were implemented on a square with sides of 100 units, with periodic boundary conditions to mimic an infinite landscape. We assumed ‘top-hat’ migration kernels and set $\delta_{P}=\delta_{S}$ which meant that migrant individuals or propagules were distributed randomly across sites within a disc of area $\pi \delta_{S}^{2}$. In uncorrelated environments, $\pi \delta_{S}^{2}$ also specifies the expected number of sites within the migration distance of a random site (Ovaskainen and Cornell, 2006a), although in correlated environments the effective number of neighbours will be somewhat higher because any focal site is itself likely to be part of an aggregation. Each simulation run was initiated by first distributing habitat sites in the landscape (as specified by the type of environment) and then simulating the host dynamics for 50 time units, a sufficient duration for the metapopulation to reach equilibrium. At this time a single susceptible population (selected at random) was infected, and $R_{*}$ was calculated using Eq. (5) where the moment terms (${\overset{̅}{S}}^{*},\Gamma_{\mathrm{SI}}^{I},\mathrm{etc}$) were computed directly from the simulated population. The results show that the approximation performs well in a variety of environments down to a migration area of ≈10 neighbouring sites (Fig. 4). For smaller migration areas (down to ≈5 neighbouring sites) the approximation still performs well in most environments, although it tends to underestimate $R_{*}.$ The approximation performs best in dynamic and correlated environments.

### 3.3 Disease invasion

If a disease does establish, the spatial structure of the metapopulation will influence both the speed of spread and the final prevalence of the disease across sites.

#### 3.3.1 Local disease migration reduces the rate of spread of the disease

Any disease that spreads by infecting local populations (either by the dispersal of pathogen propagules or by the movement of infected hosts) will tend swiftly to colonise locally available sites. As these become infected, and further susceptible sites are less accessible, the rate of disease spread falls.

**Fig. 5:**
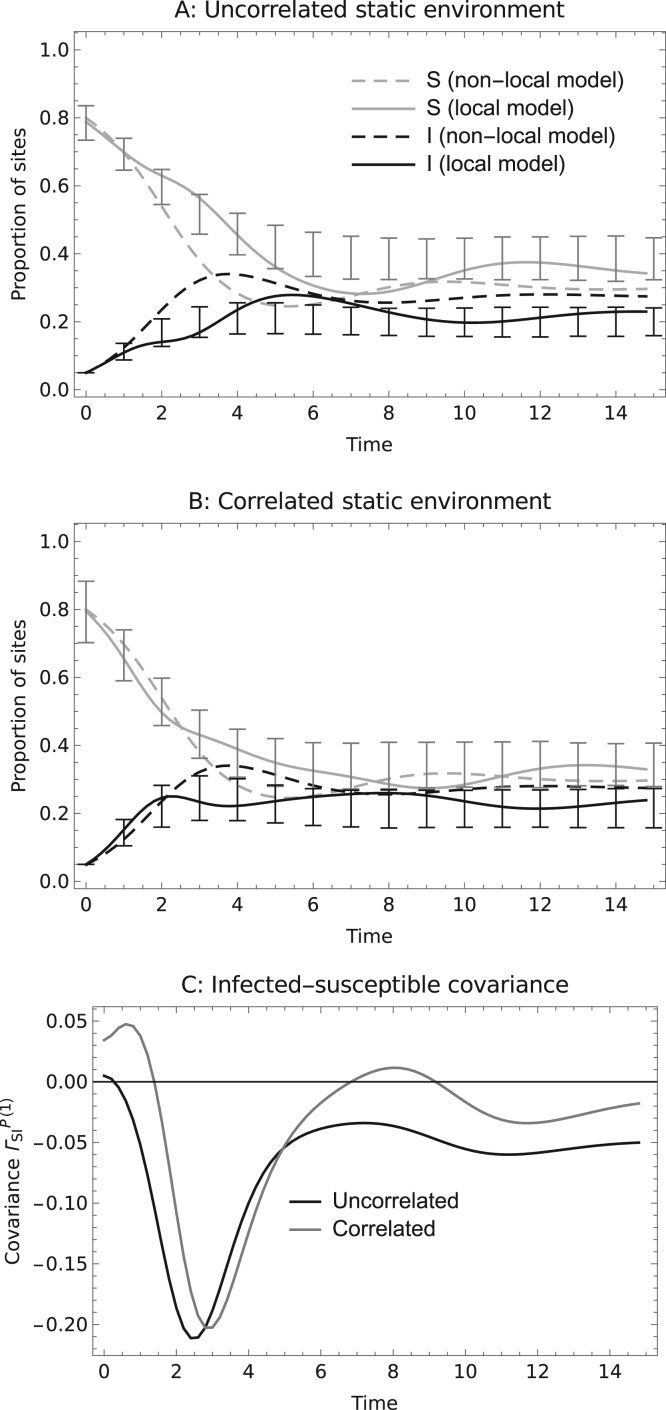
The dynamics of an invasion in an uncorrelated static environment (A; ν≈0) and a correlated static environment (B; ν=4,λ=0.5). We assume the disease spreads by the dispersal of pathogen propagules, with parameters so that invasion is likely ($m_{S}=2,m_{I}=0,m_{P}=2,\mu_{S}=0.3,\mu_{I}=0.6$ giving $R_{*}^{\left(0\right)}=2.753$). In the upper plots, the solid lines plot the dynamics derived from the first order perturbation approximation, the dashed lines plot the dynamics from the equivalent non-local model and the bars plot the mean and standard errors of simulation results. The lower plot shows the covariance between susceptible and infected populations (${\Gamma_{\mathrm{SI}}^{P}}^{\left(1\right)}$) for the two invasion scenarios. The dispersal kernels are top-hat and the landscape kernel is Gaussian.

Consider a disease that spreads by the dispersal of pathogen propagules. The disease enters the metapopulation by infecting a susceptible site and where the spatial ecology of the host population has resulted in the aggregation of occupied sites (see above) $\Gamma_{\mathrm{SI}}$ is thus initially positive, more so in correlated compared with uncorrelated environments. In uncorrelated environments, $\Gamma_{\mathrm{SI}}$ quickly becomes negative as local susceptible sites are infected and the rate of spread of the disease drops below that of the equivalent non-spatial model (Fig. 5A). In correlated environments the switch from positive to negative covariance is slower (Fig. 5B).

If the disease spreads by the movement of infected hosts, then the initial infection is at a random site and $\Gamma_{\mathrm{SI}}$ is initially small and very quickly becomes negative, reducing the rate of disease spread. However, if the disease does not severely affect colonisation (i.e. if $m_{I}\approx m_{S}$), the effect is less pronounced because then the proximity to susceptible populations is less important. Moreover, if the environment is correlated, such a disease may spread faster than predicted by the non-spatial theory due to colonisation rates being higher where empty sites are aggregated.

#### 3.3.2 Spatial structure may affect the equilibrium prevalence of the disease

If a disease is able to invade the metapopulation, its frequency will increase either to the point where all occupied sites are infected or until an equilibrium is reached where infected and uninfected sites coexist, the two scenarios being called ‘pandemic’ and ‘endemic’ by Hess (1996). In the absence of local spatial processes we can derive a threshold condition for the disease to become pandemic,

(7) $$bm_{I}+m_{P}>\frac{\beta \left(m_{S}-m_{I}\right)+\mu_{I}m_{S}-\mu_{S}m_{I}}{m_{I}-\beta -\mu_{I}},$$

which generalises a similar condition derived by Hess (1996) for a limiting case of our non-local model (where $m_{P}=\beta =0$ and $m_{S}=m_{I}$).

The pandemic state is more likely for diseases that impose only a modest fitness cost on their host ($m_{I}\approx m_{U},\mu_{I}\approx \mu_{U}$) yet are highly infectious (high *b* or $m_{P}$). Once it is reached and there are only two types of site (empty and occupied) the properties of the metapopulation can be derived from the disease-free theory after making adjustments for the effect of the disease. In particular we expect the disease to decrease dispersal and increase occupied site extinction rates which will tend to reduce the equilibrium frequency of occupied sites. Similarly the effect of local dispersal and environmental correlations on the structure of pandemic metapopulations can be deduced from the theory for disease-free metapopulations described above.

**Fig. 6:**
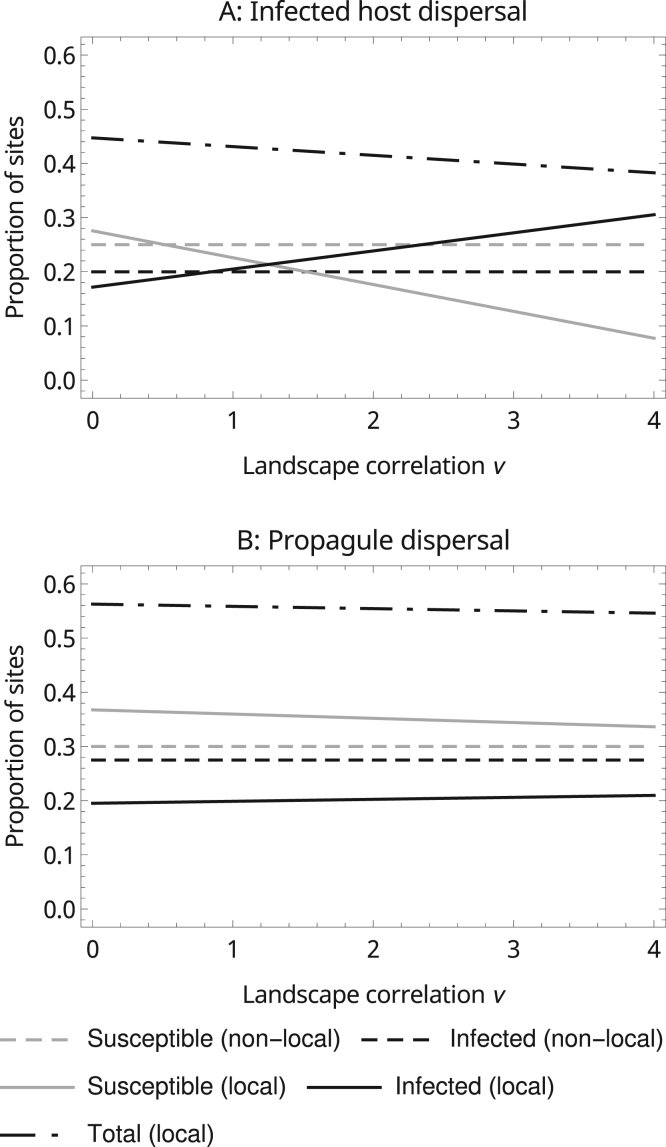
The effect of environmental correlation on the equilibrium densities of infected and susceptible populations. Parameters are A: $m_{S}=1,m_{I}=0.75,b=1,\mu_{S}=0.4,\mu_{I}=0.6,\lambda =0.5$; B: $m_{S}=2,m_{I}=0,m_{P}=2,\mu_{S}=0.3,\mu_{I}=0.6,\lambda =0.5$. The dispersal and landscape kernels are all Gaussian.

An endemic equilibrium will result if the disease is able to establish but the pandemic threshold condition is not met. We investigated the influence of local dispersal on the long-term structure of endemic metapopulations by solving the equilibrium expressions up to the first order of the perturbation expansion (Eq. (2a–c) after setting the derivatives to zero). The same spatial factors which influence the rate of disease spread also influence its equilibrium structure. First, because susceptible sites near infected sites are more likely to be infected, a degree of spatial segregation obtains at the endemic equilibrium (${\Gamma^{*}}_{\mathrm{SI}}<0$), which leads to lower disease prevalence in comparison with the non-local case. Second, we found that environmental correlation tends to lead to higher disease prevalence by increasing the local connectivity of the metapopulation. Which of these two processes has the greatest effect on disease prevalence depends on disease biology. In the case of a disease that spreads by the movement of infected hosts and which imposes only a modest reduction on the ability of the host to colonise empty habitat sites (e.g. in Fig. 6A where $m_{I}=0.75m_{U}$) the second is the most important. If the disease spreads by dispersing pathogen propagules then the effect of the negative covariance between host and disease is likely to dominate so that spatial factors reduce the equilibrium prevalence of disease (Fig. 6B).

## 4 Discussion

If the individuals in a metapopulation tend to disperse locally, the metapopulation will come to have a spatial distribution in which occupied sites are aggregated. Our results demonstrate that this spatial structure may both facilitate the establishment of a pathogen and hinder its ability to persist in the population. Since establishment and persistence are both necessary for a pathogen to form an epidemic, the overall role of host aggregation will depend on the relative importance of each factor in any particular situation. We investigated how the relative roles of these two processes are influenced by the host dispersal kernel, the transmission biology of the invading pathogen, and the spatial distribution of the populations that constitute the metapopulation.

The effects of spatial structure are greatest when host dispersal is short-range, and where the underlying environment is correlated and static, because these are the conditions that give rise to the most aggregated host metapopulations. For a given metapopulation, the effects of host aggregation in both facilitating establishment and hindering persistence are most pronounced for pathogens that (i) disperse between host populations independently of host movements and (ii) disperse over short spatial scales. The effects of spatial structure are weaker in the case of pathogens that are transmitted between populations by the movement of infected hosts and so are able to spread by the colonisation of empty habitat (with their host) as well as by the infection of susceptible populations. This type of pathogen can enter an environment by the colonisation of an empty site, which will not on average be particularly near to susceptible populations. On the other hand, an invasion will be less affected by a build-up of negative covariance between infected and susceptible host populations if the pathogen can spread by colonisation of empty habitats as well as by infection of susceptible populations.

Our conclusions on the mixed influence of host dispersal may appear somewhat at odds with models that have suggested that host populations with greater dispersal are more susceptible to disease invasion due to the movement of infected individuals to uninfected parts of the landscape (Hess, 1996, Gog et al., 2002, McCallum and Dobson, 2002, Cross et al., 2005, Cross et al., 2007, Colizza and Vespignani, 2008, Jesse et al., 2008, Harding et al., 2012). There are two reasons for this apparent disparity.

First, in our model the spatial structure of the susceptible metapopulation is an emergent property of the host and landscape characteristics. Previous models of the spatial spread of pathogens in metapopulations have mostly been motivated by human pathogens for which the spatial structure of the metapopulation is essentially independent of host movements (e.g. Hufnagel et al., 2004; Cross et al., 2005, Cross et al., 2007; Colizza and Vespignani, 2008). Our approach is more appropriate for classical metapopulations characterised by frequent extinctions and colonisations (Hanski and Gaggiotti, 2004). Perhaps the clearest examples are those of insect host metapopulations that are attacked by specialist hymenopteran parasitoids. Parasitoids are insects whose larvae feed on a single host that they eventually kill, but which have free-living and dispersive adults. Their biology is equivalent to a pathogen that disperses independently of its host. The best-studied example is the butterfly *Melitaea cinxia* attacked by *Cotesia melitaearum* and *Hyposoter horticola* (Lei and Hanski, 1997, Lei and Hanski, 1998). The host population structure is a classical metapopulation and the presence of the parasitoid in a population increases the risk of its extinction. Of the two parasitoids attacking *M. cinxia* in a Finnish metapopulation, Lei and Hanski (1998) found that one species, *C. melitaearum* tends to occur in well connected host populations where it out-competes the other species *H. horticola*, while *H. horticola* is more common in isolated host populations due to its longer dispersal range. Our results suggest that the shorter-range dispersal of *C. melitaearum* may contribute to its competitive advantage in well-connected populations, while the longer-range dispersal of *H. horticola* may lead to its population being more stable over time (Lei and Hanski, 1998).

Second, we have focussed on dispersal range (how far do dispersers move?), rather than dispersal rate (how many dispersers?) which has previously been the focus of most spatially implicit metapopulation models (Hess, 1996, McCallum and Dobson, 2002, Gog et al., 2002, Jesse et al., 2008, Harding et al., 2012) and lattice models (Cross et al., 2005, Cross et al., 2007). Hess (1996) showed that increasing the dispersal rate invariably increases the likelihood of disease invasion due to both more movement of infected individuals to susceptible populations, and more colonisation of unoccupied habitat. Hess's model can be interpreted as a limiting case of our model as dispersal becomes non-local and we recover the same results depending on which dispersal rate is increased (susceptible hosts, infected hosts, or pathogen propagules). Our focus on dispersal range has allowed us to extend this result to examine the explicit role of spatial structure.

Our results suggest that pathogens which spread independently of their host are more likely to become established if they show short-range dispersal (Fig. 1) which is in contrast to the results of Schreiber and Lloyd-Smith (2009) who studied the role of dispersal rate in disease establishment in a finite metapopulation, where the patches vary in both quality and connectedness. By deriving a spatially averaged reproductive number $\hat{R}$, they found that dispersal promotes invasion if $\hat{R}>1$ because it increases the chance that some propagules are introduced to the best habitat patches (a form of bet-hedging). If $\hat{R}<1$ the invader may still establish if the introduction site happens to be in a locally good region (where $R_{0}>1$), but only if the dispersal rate is not so high as to diminish the influence of local habitat quality; an intermediate dispersal rate is found to be optimal for the pathogen in this case. A similar conclusion was obtained by Brown and Bolker (2004), who studied disease invasion in plant populations. Using a spatially-explicit individual-based model, they found that invasion is most likely for moderate pathogen propagule dispersal ranges, since this balances the advantage of local establishment given by short dispersal with the ability to spread to other parts of the landscape which is favoured by longer dispersal.

Our model produces different results because short-range dispersal enables a disease to take advantage of the aggregated structure of a host population, a process that has previously been described by ecologists in the context of population growth in landscapes with spatial covariance in habitat quality (*habitat association*, Bolker, 2003; *growth-density covariance*, Snyder and Chesson, 2003; *spatial inheritance*, Schauber et al., 2007). This factor is responsible for the advantage to intermediate over long-range dispersal in the models of Brown and Bolker (2004) and Schreiber and Lloyd-Smith (2009). In our case, it applies because we assume the initial invasion site is a susceptible population, which is analogous to assuming the introduction site is a good habitat. We do not see the effect of bet-hedging in our model because of our focus on dispersal range rather than dispersal rate: the reduced ability of less dispersive diseases to infect distant populations is balanced by an increased ability to infect local populations.

If a disease does establish in a metapopulation, its spread may be impeded by a build-up of negative covariance between infected and susceptible host populations (Fig. 5). A similar effect, acting at the level of individuals, has been observed in network models (Keeling, 1999, 2005; Eames, 2008) and spatially explicit models of plant populations (Bolker, 1999, Brown and Bolker, 2004). Its potential to slow or prevent epidemics led Keeling (1999) to suggest that the basic reproduction number should be redefined as the number of secondary infections per case once the early spatial pattern has formed and equilibrated, an approach followed by Brown and Bolker (2004) as well as by Keeling himself (Keeling, 1999). We agree that to evaluate the risk of diseases invading metapopulations, more attention should be given to $R_{*}$, the population level equivalent of $R_{0}$ (Ball et al., 1997, Cross et al., 2005, Cross et al., 2007). In classical metapopulations, where all populations are assumed to be equally connected, knowledge of $R_{*}$ is sufficient to predict the risk of an epidemic occurring. Here we have shown, however, that the effect of negative covariance in impeding invasion at an individual level also applies at the level of local populations if these too are spatially structured. Thus there may be scenarios where $R_{*}>1$, yet the disease does not spread from rare. If $R_{*}<1$, our numerical analysis indicates that a build-up of spatial covariance will not assist establishment for example if a large number of early cases arise by chance, because this spatial covariance acts to reduce the disease spread rate. We thus conjecture that a disease will not persist if $R_{*}<1$, although we have not proved this mathematically, so that $R_{*}>1$can be viewed as a conservative condition of the short term establishment of a disease rather than its long term spread.

Since establishment is a necessary precursor of spread, we consider $R_{*}$ is nevertheless a useful measure of disease risk in metapopulations - by defining a boundary below which diseases will be unable to invade ($R_{*}<1$). It is hoped that future work will develop a more general ‘invasion rate of metapopulations’ metric $R_{I}>R_{*}$, that enables diseases that can establish but not spread to be distinguished from those that will spread persistently (i.e. a metapopulation equivalent to the metrics developed by Keeling, 1999 and Brown and Bolker, 2004). In theory, the leading eigenvalue of the coupled dynamics of infected and uninfected populations will give this information, yet we have had difficulty computing this measure due to the non-convergence of an integral in parts of the parameter space. However, our results highlight the significance of this issue.

Note that in finite populations, metrics such as $R_{*}$ (or an invasion eigenvalue) do not fully predict whether a disease will establish or invade, but rather help determine the probabilistic outcome. For example in the simplest non-spatial epidemic models the probability of invasion is $1-{\left(\frac{1}{R_{0}}\right)}^{I_{0}}$ if $R_{0}>1$ (and 0 otherwise), where $I_{0}$ is the initial number of infected individuals (Diekmann and Heesterbeek, 2000). Therefore $R_{0}>1$ can be viewed as a condition for invasion *provided there is a large introduction of disease*. In a finite version of our spatially structured metapopulation, $R_{*}>1$ can similarly be viewed as a condition for long term persistence of a disease, but not necessarily its spread, given a large number of populations are initially infected. To illustrate and justify this definition, the supplementary Fig. S1 plots the probability that a disease persists for at least 100 time units against infectiousness, for a disease that spreads by the dispersal of pathogen propagules, in both uncorrelated and correlated static landscapes (computed from simulations). We see that the first order approximation of $R_{*}$ is a good indicator of the threshold above which persistence is possible following large introductions. In a very few simulations, the disease persisted despite $R_{*}<1$, which may be due to the finite simulated duration or due to higher order spatial effects not incorporated in the first order approximation.

In conclusion, we have demonstrated that factors which influence the spatial structure of a metapopulation in the absence of a disease will also influence the probability of its invasion by a pathogen. Our results also emphasize the importance of the mean rate and range of pathogen dispersal, when this occurs independently of the host, in understanding establishment and spread. Although we have focussed on idealised scenarios, the modelling framework we have developed is flexible and can be extended, for example by including local population dynamics or variation in the quality of habitat sites. The choice of our modelling framework also facilitates the incorporation of data from real metapopulations which often is restricted to the presence or absence of healthy and diseased populations at each site (Moilanen, 1999, Etienne et al., 2004). We hope that this link with data will allow further study of the factors that determine disease invasions in natural populations and improve our ability to manage them.

## Figures and Tables

**Table 1:** Transition rates amongst classes of site.

Transition	Rate
*E*→*S*	$m_{S}\sum_{i\in S}D_{S}\left(\rho_{i}\right)$
*E*→*I*	$m_{I}\sum_{i\in I}D_{I}\left(\rho_{i}\right)$
*S*→*E*	*μ*_*S*_
*S*→*I*	$m_{I}b\sum_{i\in I}D_{I}\left(\rho_{i}\right)+m_{P}\sum_{i\in I}D_{P}\left(\rho_{i}\right)$
*I*→*E*	*μ*_*I*_

## References

[bib1] Ball F., Mollison D., Scalia-Tomba G. (1997). Epidemics with two levels of mixing. Ann. Appl. Probab..

[bib2] Bolker B. (2003). Combining endogenous and exogenous spatial variability in analytical population models. Theor. Popul. Biol..

[bib3] Bolker B., Pacala S.W. (1997). Using moment equations to understand stochastically driven spatial pattern formation in ecological systems. Theor. Popul. Biol..

[bib4] Bolker B.M. (1999). Analytic models for the patchy spread of plant disease. Bull. Math. Biol..

[bib5] Brown D.H., Bolker B.M. (2004). The effects of disease dispersal and host clustering on the epidemic threshold in plants. Bull. Math. Biol..

[bib6] Clobert J., Ims R., Rousset F., Hanski I., Gaggiotti O. (2004). Causes, mechanisms and consequences of dispersal. Ecology, Genetics, and Evolution of Metapopulations.

[bib7] Colizza V., Vespignani A. (2008). Epidemic modeling in metapopulation systems with heterogeneous coupling pattern: theory and simulations. J. Theor. Biol..

[bib8] Cornell S.J., Ovaskainen O. (2008). Exact asymptotic analysis for metapopulation dynamics on correlated dynamic landscapes. Theor. Popul. Biol..

[bib9] Cross P.C., Lloyd-Smith J.O., Johnson P.L., Getz W.M. (2005). Duelling timescales of host movement and disease recovery determine invasion of disease in structured populations. Ecol. Lett..

[bib10] Cross P.C., Johnson P.L., Lloyd-Smith J.O., Getz W.M. (2007). Utility of *R*_0_ as a predictor of disease invasion in structured populations. J. R. Soc. Interface.

[bib11] Diekmann O., Heesterbeek J. (2000). Mathematical Epidemiology of Infectious Diseases: Model Building, Analysis and Interpretation.

[bib12] Eames K.T. (2008). Modelling disease spread through random and regular contacts in clustered populations. Theor. Popul. Biol..

[bib13] Etienne R.S., ter Braak C.J., Vos C.C., Hanski I., Gaggiotti O. (2004). Application of stochastic patch occupancy models to real metapopulations. Ecology, Genetics, and Evolution of Metapopulations.

[bib14] Ferguson N.M., Donnelly C.A., Anderson R.M. (2001). The foot-and-mouth epidemic in great britain: pattern of spread and impact of interventions. Science.

[bib15] Gog, J., Woodroffe, R., Swinton, J., 2002. Disease in endangered metapopulations: the importance of alternative hosts. In: Proceedings of the Royal Society of London, Series B: Biological Sciences, 269, 1492, pp. 671–676 10.1098/rspb.2001.1667 PMC1690941 11934357

[bib16] Hanski I., Gaggiotti O., Hanski I., Gaggiotti O. (2004). Metapopulation biology: past, present, and future. Ecology, Genetics, and Evolution of Metapopulations.

[bib17] Harding K., Begon M., Eriksson A., Wennberg B. (2012). Increased migration in host–pathogen metapopulations can cause host extinction. J. Theor. Biol..

[bib18] Hess G. (1996). Disease in metapopulation models: implications for conservation. Ecology.

[bib19] Hufnagel, L., Brockmann, D., Geisel, T., 2004. Forecast and control of epidemics in a globalized world. In: Proceedings of the National Academy of Sciences of the United States of America, 101, 42, pp. 15124–15129 10.1073/pnas.0308344101 PMC524041 15477600

[bib20] Jesse M., Ezanno P., Davis S., Heesterbeek J. (2008). A fully coupled, mechanistic model for infectious disease dynamics in a metapopulation: movement and epidemic duration. J. Theor. Biol..

[bib21] Keeling M.J., Woolhouse M.E., Shaw D.J., Matthews L., Chase-Topping M., Haydon D.T., Cornell S.J., Kappey J., Wilesmith J., Grenfell B.T. (2001). Dynamics of the 2001 UK foot and mouth epidemic: stochastic dispersal in a heterogeneous landscape. Science.

[bib22] Keeling, M.J., 1999. The effects of local spatial structure on epidemiological invasions. In: Proceedings of the Royal Society of London, Series B: Biological Sciences, 266, 1421, pp. 859–867 10.1098/rspb.1999.0716 PMC1689913 10343409

[bib23] Keeling M. (2005). The implications of network structure for epidemic dynamics. Theor. Popul. Biol..

[bib24] Lei G., Hanski I. (1998). Spatial dynamics of two competing specialist parasitoids in a host metapopulation. J. Anim. Ecol..

[bib25] Lei G.-C., Hanski I. (1997). Metapopulation structure of *Cotesia melitaearum*, a specialist parasitoid of the butterfly *Melitaea cinxia*. Oikos.

[bib26] Levins R. (1969). Some demographic and genetic consequences of environmental heterogeneity for biological control. Bull. ESA.

[bib27] McCallum, H., Dobson, A., 2002. Disease, habitat fragmentation and conservation. In: Proceedings of the Royal Society of London, Series B: Biological Sciences, 269, 1504, pp. 2041–2049 10.1098/rspb.2002.2079 PMC1691124 12396504

[bib28] Moilanen A. (1999). Patch occupancy models of metapopulation dynamics: efficient parameter estimation using implicit statistical inference. Ecology.

[bib29] Ovaskainen O., Cornell S.J. (2006). Asymptotically exact analysis of stochastic metapopulation dynamics with explicit spatial structure. Theor. Popul. Biol..

[bib30] Ovaskainen, O., Cornell, S.J., 2006b. Space and stochasticity in population dynamics. In: Proceedings of the National Academy of Sciences of the United States of America, 103, pp. 12781–12786 10.1073/pnas.0603994103 PMC1568924 16912114

[bib31] Schauber E.M., Goodwin B.J., Jones C.G., Ostfeld R.S. (2007). Spatial selection and inheritance: applying evolutionary concepts to population dynamics in heterogeneous space. Ecology.

[bib32] Schreiber S.J., Lloyd-Smith J.O. (2009). Invasion dynamics in spatially heterogeneous environments. Am. Nat..

[bib33] Snyder R., Chesson P. (2003). Local dispersal can facilitate coexistence in the presence of permanent spatial heterogeneity. Ecol. Lett..

[bib34] Watts, D.J., Muhamad, R., Medina, D.C., Dodds, P.S., 2005. Multiscale, resurgent epidemics in a hierarchical metapopulation model. In: Proceedings of the National Academy of Sciences of the United States of America, 102, 32, pp. 11157–11162 10.1073/pnas.0501226102 PMC1183543 16055564

