# Supplementary material for: The dynamics of disease in a metapopulation: The role of dispersal range
Source: J Theor Biol. 2017 Apr 7;418:57–65. doi: 10.1016/j.jtbi.2017.01.037 (PMC5360276; doi:10.1016/j.jtbi.2017.01.037)
Supplement: Supplementary file 1 — Supplementary material [file mmc1.pdf]

Supplementary Online Material for *The dynamics of disease in a metapopulation: the role of dispersal range*

Here we derive the equations of the first spatial moment (eqn. 2 in the main text), and its first order perturbation expansion. The general procedure closely follows that in previous papers, in particular Ovaskainen and Cornell (2006) and Cornell and Ovaskainen (2008).

Derivation of the moment equations

First, note that the sets $S(t), I(t)$ and $E(t)$ are sets of *points* in 2-dimensional space and so can be described as sums of delta-distributions, e.g.

$$S(x,t) = \sum_i \delta(x - x_i)$$

where x_i is the location of the ith susceptible population (subsequently we will write $S(x,t) = S$ etc. where the space and time dependence is clear). The dynamics of the three sets are fully described by the transition rates given in the sections "Site dynamics" and "Site type transition rates" in the main text. For the derivation it is convenient to write them as a system of stochastic differential equations,

$$\begin{aligned} dS &= \Big[m_S(D_S * S)E - m_I b(D_I * I)S + m_P(D_P * I)S \\ &\quad -(\mu_S + \beta)S\Big]dt + d\eta_S \qquad (1) \\ dI &= \Big[m_I(D_I * S)E + m_I b(D_I * I)S + m_P(D_P * I)S \\ &\quad -(\mu_I + \beta)I\Big] + d\eta_I \qquad (2) \\ dQ &= \Big[\alpha\nu - \beta Q\Big] + d\eta_Q, \qquad (3) \end{aligned}$$

where $Q = S + I + E$ is the set of all sites, $*$ denotes convolution and the terms of the form $d\eta_X$ represent the stochastic noise that is generated by the underlying stochasticity of the site dynamics. By definition, the expectation of this noise is zero.

To transition from the individual-based description of the model to the population based moment-equations, we first decompose the state variables into the first spatial moment and residual, for example

$$S(x,t) = \bar{S}(t) + S_s(x,t) \qquad (4)$$

where $\bar{\cdot}$ denotes expectation over realisations of the underlying model and $\cdot_s(x,t)$ represents the stochastic fluctuation around this (i.e. $\overline{S_s(x,t)} = 0$). We will also need the second spatial moments $G_{XY}(\rho, t)$, which are defined as, for example,

$$G_{SE}(\rho = |x_1 - x_2|, t) = \overline{S_s(x_1,t)E_s(x_2,t)}.$$

The dynamics of the first spatial moments

To derive ordinary differential equations for the dynamics of the first spatial moments (eqn. 2 in the main text), we substitute the first moment decompositions (eqn. 4) into equations 1-3 above, and take expectations of each side. For example,

$$\begin{aligned}
\frac{\overline{dS}}{dt} &= \frac{d\overline{S}}{dt} \\
&= \overline{\Big[m_S(D_S*(\bar{S}+S_s))(\bar{E}+E_s) - m_I b(D_I*(\bar{I}+I_s))(\bar{S}+S_s)} \\
&\quad \overline{-m_P(D_P*(\bar{I}+I_s))(\bar{S}+S_s) - (\mu_S+\beta)(\bar{S}+S_s)\Big]}dt + \frac{\overline{d\eta_S}}{dt} \\
&= \underbrace{\Big[m_S\overline{SE} - m_I b\overline{SI} - m_P\overline{SI} - (\mu_S+\beta)\overline{S}\Big]}_{\text{independent of spatial structure}} \\
&\quad \underbrace{+\Big[m_S\overline{(D_S*S_s)E_s} - m_I b\overline{(D_I*I_s)S_s} - m_P\overline{(D_P*I_s)S_s}\Big]}_{\text{contribution of spatial structure}}. \qquad (5)
\end{aligned}$$

This is equivalent to the the $d\bar{S}/dt$ component of eqn. 2 in the main text, because the covariance terms such as $\overline{(D_S*S_s)E_s}$ are equivalent to Γ terms, in this example Γ^S_{SE}, as defined in the main text (eqn. 1). This follows from expanding the convolution,

$$\begin{aligned}
\overline{(D_S*S_s)E_s} &= \overline{\int D_S(y)S_s(x-y)dyE_s(x)} \\
&= \int D_S(y)G_{SE}(y)dy \\
&= \Gamma^S_{SE}.
\end{aligned}$$

The $\frac{d\bar{I}}{dt}$ and $\frac{d\bar{Q}}{dt} = \frac{d\overline{(S+I+E)}}{dt}$ terms of eqn. 2 in the main text are similarly derived.

The dynamics of the second spatial moments

Before applying the perturbation expansion, we need to derive equations for the dynamics of the second moment terms (G_{ij}) from which we can calculate the Γ terms. We can derive expressions for the evolution of the covariance terms, for example

$$\begin{aligned}
dG_{SI}(x,t) &= G_{SI}(x,t+\delta t) - G_{SI}(x,t) \\
&= \overline{(S_s+dS_s)(I_s+dI_s)} - \overline{S_sI_s} \\
&= \overline{dS_sI_s} + \overline{S_sdI_s} + \overline{dS_sdI_s} \\
\Rightarrow dG_{ij}(x,t) &= \overline{di_sj_s} + \overline{i_sdj_s} + \overline{di_sdj_s} \quad (i,j \in \{S,I,Q\}) \qquad (6)
\end{aligned}$$

These equations can be expanded by extracting the residual stochastic parts of the original stochastic differential equations (eqns 1-3). The expanded expressions fast become complex and so we give a representative sample here rather than the full derivations. The derivative $\frac{dG_{SS}}{dt}(x,t)$ contains terms that stem from the colonisation of empty sites by susceptible populations. In equation 1 this is represented by the term $m_S(D_S*S)Edt$. The expansion of $dG_{SS}(x,t)$ thus contains the terms

$$2m_S\left(\overline{[(D_S*S_S)\bar{E} + \bar{S}E_S + (D_S*S_S)E_s]S_s}\right)dt \qquad (7)$$

which come from expanding the first two terms on the right hand side of eqn. 6. The third term is the covariance of noise associated with this event (written $\overline{d\eta_S d\eta_S}_{\text{colonisation}} = \overline{d\eta_S d\eta_S}$ due to this kind of infection). This is derived by considering a small region of size dx around the point x, where dx is so small that it contains at most one site. In the small time interval dt we have

$$\begin{aligned}\overline{d\eta_S d\eta_S}_{\text{colonisation}}(x) &= \underbrace{(-1/dx)^2}_{\text{change to } S} \times \overline{\underbrace{E(x)dx}_{\text{region has empty site}} \times \underbrace{m_S(D_S * S)(x)dt}_{\text{it is colonised during } dt}} \\ &= m_s\overline{(D_S * S)E}\delta(x)dt \quad \text{(in the limit of } dx \to 0). \end{aligned} \tag{8}$$

Combining eqns. 7 and 8, we find that $\frac{dG_{SS}}{dt}(x,t)$ contains the terms

$$m_s\left(2\bar{E}D_S * G_{SS} + 2\bar{S}G_{SE} + 2D_S * H_{SSE} + \delta(x)(\bar{S}\bar{E} + D_S * G_{SE})\right) \tag{9}$$

where the triplet term H_{SSE} is a third spatial moment. We note that the second moment $G_{SS}(x,t)$ contains a delta peak because of the 'self-covariance' of a given site,

$$G_{SS}(x,t) = \overline{S_s(x,t)S_s(x,t)} = \bar{S}\delta(x).$$

It is convenient to take a final step of removing all δ peaks from the covariance terms G_{ij}, by defining the smooth part of the matrix G as G^*,

$$G^*(x) = G(x) - \Pi\delta(x)$$

where $\Pi(x)$ is the diagonal matrix

$$\Pi(x) = \begin{pmatrix} \bar{S}, 0, 0 \\ 0, \bar{I}, 0 \\ 0, 0, \bar{E} \end{pmatrix}. \tag{10}$$

Substituting the smoothed version of G into eqn. 9 removes the delta term from the derivative, so that

$$\begin{aligned}\frac{dG^*_{SS}}{dt}(x,t) &= \frac{dG^*_{SS}}{dt}(x,t) - \frac{d\bar{S}}{dt}(x,t)\delta(x) \\ &= m_s\Big(2\bar{E}D_S * (G^*_{SS} + \bar{S}\delta(x)) + 2\bar{S}G^*_{SE} + 2D_S * H_{SSE} \qquad (11)\\ &\quad +\delta(x)(\bar{S}\bar{E} + D_S * G^*_{SE}) \\ &\quad -(\bar{S}\bar{E} + D_S * G_{SE})\delta(x)\Big) \\ &\quad + \cdots \text{[terms from other processes e.g. infection]} \\ &= 2m_s(\bar{E}(D_S * G^*_{SS} + \bar{S}D_S) + \bar{S}G^*_{SE} + D_S * H_{SSE}) + \cdots \qquad (12)\end{aligned}$$

Perturbation expansion

The essence of the perturbation expansion is to redefine each spatial moment as power-series, such as equation 3 in the main text,

$$\overline{S}(t) = \overline{S}(t)^{(0)} + \epsilon\overline{S}(t)^{(1)} + \epsilon^2\overline{S}(t)^{(2)} + \cdots,$$

and collect terms of equal order. It can be shown (Cornell and Ovaskainen, 2008) that the nth contains no terms of order $n-2$, so we have

$$G^*_{ij} = \epsilon G^{(1)^*}_{ij} + \epsilon^2 G^{(2)^*}_{ij} + \cdots,$$
$$H^*_{ijk} = \epsilon^2 H^{(2)^*}_{ijk} + \cdots etc.$$

The mean field behaviour of the model can thus be studied by ignoring the second (G) and higher moments, and the first order solution can be studied by ignoring H and the higher moments. Due to the convolutions in the equations of the second moment dynamics, it is convenient to transform these equations into Fourier space (this converts convolutions into products).

As stated in the main text, the perturbation parameter ϵ inversely scales the widths of the environment and dispersal kernels in the model, by defining each of the kernel scales via an implicit parameter, $((\delta_S, \delta_I, \delta_P, \lambda) = (\delta'_S, \delta'_I, \delta'_P, \lambda')/\sqrt{\epsilon})$.

Expansion of the first moment

Mean field ('zeroth order')

At the limit of $\epsilon \to 0$, the spatial kernels become infinitely broad so that the spatial structure of the metapopulation becomes irrelevant to the metapopulation dynamics. This is the "mean-field" limit and metapopulation models of this type are sometimes called "spatially implicit" or "classical". Since the covariance terms (G) are zero in this limit, these can be ignored to obtain the spatially implicit version of this model from equation 1 and the others like it. This is given by

$$\frac{d\overline{S}^{(0)}}{dt} = m_S \overline{S}^{(0)} \overline{E}^{(0)} - m_I b \overline{S}^{(0)} \overline{I}^{(0)} - m_P \overline{S}^{(0)} \overline{I}^{(0)} - (\mu_S + \beta)\overline{S}^{(0)} \quad (13)$$

$$\frac{d\overline{I}^{(0)}}{dt} = m_I \overline{I}^{(0)} \overline{E}^{(0)} + m_I b \overline{S}^{(0)} \overline{I}^{(0)} + m_P \overline{S}^{(0)} \overline{I}^{(0)} - (\mu_I + \beta)\overline{I}^{(0)} \quad (14)$$

$$\frac{d\overline{Q}^{(0)}}{dt} = \alpha\nu - \beta\overline{Q}^{(0)} \quad (15)$$

First order

The first order equations are derived by substituting all terms up to first order into the first moment equations (eqn. 1 etc), and collecting the first order parts. For example, equation 1 becomes

$$\epsilon\frac{d\overline{S}^{(1)}}{dt} = \epsilon\Big[m_S(\overline{S}^{(0)}\overline{E}^{(1)} + \overline{S}^{(1)}\overline{E}^{(0)}) \quad (16)$$
$$-m_I b(\overline{S}^{(0)}\overline{I}^{(1)} + \overline{S}^{(1)}\overline{I}^{(0)}) \quad (17)$$
$$-m_P(\overline{S}^{(0)}\overline{I}^{(1)} + \overline{S}^{(1)}\overline{I}^{(0)}) - (\mu_S + \beta)\overline{S}^{(1)}$$
$$+m_S\Gamma^{S^{(1)}}_{SE} - m_I b\Gamma^{I^{(1)}}_{SI} - m_P\Gamma^{P^{(1)}}_{SI}\Big] \quad (18)$$

Expansion of R_*

The exact expression for R_* is given in the main text (eqn. 5),

$$R_* = \frac{1}{\mu_I + \beta}\left(m_I(1 - S^*) + m_I bS^* + m_P S^* + \lim_{\bar{I}\to 0}\frac{m_I\Gamma^I_{IE} + m_I b\Gamma^I_{SI} + m_P\Gamma^P_{SI}}{\bar{I}}\right). \quad (19)$$

where we have assumed that the landscape parameters (α, ν, β) are such that the average density of sites $\bar{Q}^*$ in equilibrium is one ($\bar{Q}^* = \frac{\alpha\nu}{\beta}$, this assumption is used for all the model analysis in the main text).

The Γ terms in eqn. 19 relate to how R_* depends on the location of the initial diseased populations, while the $\bar{S}^*$ terms describe the role of susceptible population size. In the mean field model, the Γ terms are zero and the mean field equation for R_* is thus

$$R_*^{(0)} = \frac{1}{\mu_I + \beta}(m_I(1 - S^{(0)*}) + m_I b S^{(0)*} + m_P S^{(0)*}). \quad (20)$$

The first order equations involve the gamma terms, yet these will depend on the initial placement of the infection. In the main text, we consider two alternatives for the initial disease placement.

(a) The disease initially occurs at a patch chosen at random among all the patches.

(b) The disease initially converts a randomly chosen susceptible population .

To implement these assumptions we suppose the initial set of infected populations is a small subset of the set of (a) all the sites ($I(0) \sim Q^*$) or (b) the susceptible populations in the pre-disease equilibrium ($I(0) \sim S^*$). In case (a) we have

$$\begin{aligned} I(0) &= \lim_{\bar{I} \to 0} \bar{I} Q \\ \Rightarrow (G_{IE}, G_{SI}) &= \lim_{\bar{I} \to 0} \bar{I}(G^*_{QE}, G^*_{SQ}) \\ \Rightarrow \lim_{\bar{I} \to 0} \frac{(\Gamma^I_{IE}, \Gamma^I_{SI}, \Gamma^P_{SI})}{\bar{I}} &= (\Gamma^I_{QE}, \Gamma^I_{SQ}, \Gamma^P_{SQ}). \end{aligned} \quad (21)$$

where we have assumed $\frac{\alpha\nu}{\beta} = Q^* = 1$.
In case (b),

$$\begin{aligned} I(0) &= \lim_{\bar{I} \to 0} \bar{I} S^* / \bar{S}^* \\ (G_{IE}, G_{SI}) &= \lim_{\bar{I} \to 0} \frac{\bar{I}}{\bar{S}^*}(G^*_{SE}, G^*_{SS}) \\ \Rightarrow \lim_{\bar{I} \to 0} \frac{(\Gamma^I_{IE}, \Gamma^I_{SI}, \Gamma^P_{SI})}{\bar{I}} &= \frac{(\Gamma^I_{SE}, \Gamma^I_{SS}, \Gamma^P_{SS})}{\bar{S}^*}. \end{aligned} \quad (22)$$

These expressions can be substituted into (19), and the first order parts are collected to obtain first order expressions for R_*,

$$R_*^{(1)} = \frac{1}{\mu_I + \beta}(m_I(1 - S^{(1)*}) + m_I b S^{(1)*} + m_P S^{(1)*}) \quad (23)$$

$$+ \begin{cases} m_I \Gamma^{I^{(0)*}}_{QE} + m_I b \Gamma^{I^{(0)*}}_{SQ} + m_P \Gamma^{P^{(0)*}}_{SQ} & \text{if (a), } I(0) \sim Q^* \\ (m_I \Gamma^{I^{(0)*}}_{SE} + m_I b \Gamma^{I^{(0)*}}_{SS} + m_P \Gamma^{P^{(0)*}}_{SS}) / (\bar{S}^{(0)*}) & \text{if (b), } I(0) \sim S^*. \end{cases} \quad (24)$$

References

Cornell, S. J. and Ovaskainen, O. (2008). Exact asymptotic analysis for metapopulation dynamics on correlated dynamic landscapes. *Theoretical population biology*, 74(3):209–225.

Ovaskainen, O. and Cornell, S. J. (2006). Asymptotically exact analysis of stochastic metapopulation dynamics with explicit spatial structure. *Theoretical Population Biology*, 69(1):13–33.
