# Supplementary material for: The dynamics of disease in a metapopulation: The role of dispersal range
Source: J Theor Biol. 2017 Apr 7;418:57–65. doi: 10.1016/j.jtbi.2017.01.037 (PMC5360276; doi:10.1016/j.jtbi.2017.01.037)
Supplement: Supplementary file 2 — Supplementary material [file mmc2.pdf]

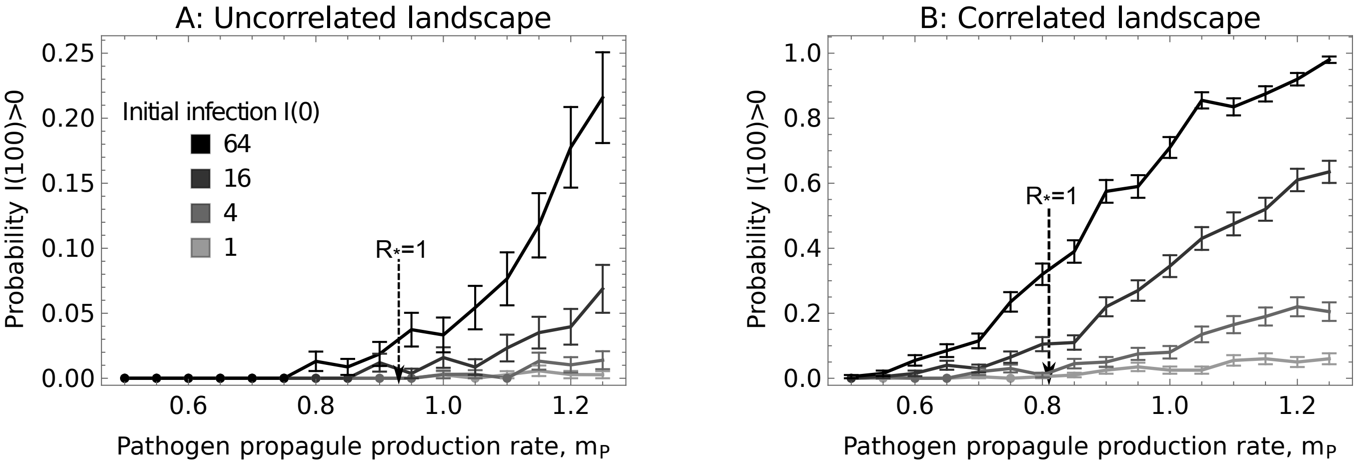

Figure S1: The probability that a disease which spreads by dispersing pathogen propagules remains in the population for 100 time units, against the propagule production rate m_p. Initially between 1 and 64 occupied patches are infected in a metapopulation with 10^5 (occupied or empty) patches. We note the zero order approximation to $R_* = 1$ is given by $m_p = 1$ while the first order approximation is $R_* = 0.93$ in the uncorrelated landscape and $R_* = 0.81$ in the correlated landscape (as shown). The parameters are as in fig. 1 with spatial scales $\delta_S = \delta_P = 2$. Results based on > 200 simulations.
